# Educational content and challenges encountered when training service user representatives as peer researchers in a mixed study on patient experience of hospital safety

**DOI:** 10.1186/s40900-020-00226-1

**Published:** 2020-09-01

**Authors:** O. Gross, N. Garabedian, C. Richard, M. Citrini, T. Sannié, R. Gagnayre

**Affiliations:** 1 Health Education and Practices Laboratory (LEPS UR3412) University Sorbonne Paris Nord, 74 rue Marcel Cachin, 93017 Bobigny, France; 2 Hôpital Necker-Enfants Malades, President of the medical board of Paris Universities Hospitals, Paris, France; 3 Hôpital Bicêtre, Member of the medical board of Paris Universities Hospitals, Le Kremlin-Bicêtre, France; 4 AP-HP service user representative, Association Créteil Respire À Coeur (Society of respiratory diseases), 38, rue des Blancs Manteaux, 75004 Paris, France; 5 AP-HP service user representative, Association française des hémophiles (French Hemophiliacs Society), 6 rue Alexandre Cabanel, 75015 Paris, France

**Keywords:** Participative research, Hospital safety, Peer interviewers, Training, Patient and public involvement

## Abstract

**Background and objectives:**

In France, following the passing of a 2002 law, service user representatives (SURs) are part of hospital committees in charge of care quality and safety issues. Ten service user representatives (SURs) were recruited and trained as “peer researchers” to participate in all phases of a study aimed at outlining how patients experience hospital safety. This article aims to describe the study protocol and how peer researchers training was designed and implemented to prepare them to drive a qualitative and quantitative research. It also examines the challenges related to collaborative research and how these were resolved.

**Methods:**

The way our training was conceived belongs to the field of “design-based research”, known for its pragmatic and collaborative scope, in which viewpoints of all participants are included. Our training was therefore based on peer researchers and research sponsors expectations, as well as on recommendations of the literature.

**Results:**

A 45-h training was held. While the program was meant to train peer researchers to respect scientific norms, it also aimed to improve their sense of self-legitimacy as they navigated their new role. Peer researchers were particularly eager to understand meaning behind the instructions, especially in the field of ethical and scientific norms. Various challenges occurred related to project organization, recruitment and peer researchers involvement. Some issues were overcome by learning how to share control over the research process.

**Conclusion:**

This experiment highlights the importance of a training program’s duration and quality to prepare SURs for their roles as peer investigators and to create a group dynamic around a research project, even with SURs familiar with patient involvement and our research theme (safety issues). Trainers overcame hurdles by being adaptive and by using educational approaches. They also learned to include trainees’ input, even when it forced them to reconsider their own assumptions.

## Plain English summary

Since 2002, French hospitals integrates service user representatives (SURs) in local committees where they are involved with care quality and safety issues. The executive committee of a group of 39 hospitals, where 139 SURs are involved, suggested them to identify a research theme they would like to see investigated. SURs proposed a study around care safety experience among hospitalized patients and offered to run it themselves. Therefore, 10 SURs were recruited to collaborate to a study outlining how patients experienced hospital safety in 10 hospitals. The study was based on the premise that SURs as peer researchers could provide unique insights as insiders, contribute to our understanding of patient experience and identify new professional practices meant to improve patient experience of hospital safety. Training and follow-up sessions took place during the research process, mainly to prepare peer-researchers to participate in all phases of a mixed study and improve their self-legitimacy to do so. Training modules were synchronized with each research step, thus allowing peer researchers to quickly apply newly acquired knowledge. Challenges emerged when peer researchers struggled to adhere to formal frameworks (inclusion criteria, interview guidelines …) and when their commitment level towards the project fluctuated. The research team overcame these obstacles by being adaptive. Training duration was longer than initially planned, mainly because peer researchers asked for more time. Even with SURs who are familiar with patient involvement and safety issues, a certain number of meetings were needed to create a positive group dynamic around the research project.

## Background

In France, principles of health democracy were instituted by the 2002 Act on Patients’ Rights and Quality of Care^1^ which includes representation of health system users. Since then, service user representatives (SURs) have been involved in all institutional agencies, including hospitals. Being nominated as SUR is subject to tightly regulated procedures. SURs must be supported by a non-profit patient/user organization with an accreditation obtained under certain conditions: proof of involvement in general interest activities linked to patient rights, financial transparency, respect of democratic rules, and assurance representativeness. These accredited organizations are, for most of them, members of a national federation, France Assos Santé. This user-led umbrella organization has four missions: monitoring of health system effective and fair operation, relaying patients/users observations and claims, informing health system users about their rights, and training user representatives in order for them to become active and relevant actors on the boards and committees where they stand, in order to improve recognition of their role. In hospitals, SURs contribute to quality of care by making sure that patients’ rights are respected, and they are particularly involved in care quality and safety issues. They fulfill this role by being statutory members of committees that address issues related to quality of care, complaints, and claims. Mediations are sometimes organized to resolve conflicts: SURs can participate in these mediations, upon agreement of the patient or his/her family. Service user representation both encompasses and fails to fully describe patient involvement and partnerships in the field of health. In the context of the Montreal model, patient partners participate in care, training, and research. This partnership model is progressively being implemented in France, in parallel with user representation as defined by the 2002 Act.

But it needs to be acknowledged that patient role recognition is still fragile in France. Unlike other countries, such as the United Kingdom for example, where paying patients for their contribution is the norm, in French hospitals (and France in general) neither SURs nor care or research patient partners receive a stipend for their contribution. And participative research initiatives are still rare.

In 2017, Paris University Hospitals (PUH) wished to associate SURs to a research study. PUH are made up of 39 hospitals located in the Île-de-France region. They serve 10 million patients per year. Within PUH, there are 139 active SURs (3 to 4 per hospital). Within the institution’s supervisory board, they are represented by two of them. PUH offered these latter to identify a research theme. Considering how adverse events are underreported by healthcare professionals [1], the fact that care safety monitoring was part of their mission and the lack of tools to evaluate patients experience around safety issues, they proposed a research program to examine how patients experienced hospital safety. They also proposed that this research be entirely conducted by SURs as peer researchers. By “peer researchers”, we mean research assistants who are recruited for the characteristics they share with study participants [2] and as active partners in research [3]. Active partners meant that they would take a large part in the research. A participative research approach, defined as made *for and by* users [4] was therefore promoted. Participative research aspires to mobilize peer researchers’ unique insights as insiders [5, 6] alongside with the knowledge and know-how of academic researchers. These research practices have developed since the 1990s [7]. In the healthcare sector, they were first implemented in the fields of disability, psychiatry and HIV infection, but have now recently expanded to a broader range of areas [8, 9].

Peer researchers can potentially participate in all phases of research as research assistants [10, 11]. Relevant training programs must therefore be designed for them to develop the required skills. However, content and objectives of the courses used to train research assistants are seldom reported, and this is particularly true for qualitative studies [12]. Training programs seem mostly focused on teaching experiential knowledge and making trainees aware of their added value (INVOLVE). But the role of a research assistant is also governed by paradoxical requirements: assistants are asked to restrict disclosure of their user/patient status [13] just as they are asked to eventually resort to it (INVOLVE); they are asked to be empathetic while also neutral [13]. Training programs should transfer power to peer researchers without overwhelming them with information [14], while also giving them a “solid understanding of basic research methods including the principles of protecting the rights and welfare of research subjects” [15]. The benefits of using these particular researchers’ profiles could be curtailed by these contradictory elements. It is therefore important to articulate a clear position. As previous studies have rarely provided details on the conception and content of these types of training, we chose to focus on the ways in which our training program was designed and implemented to prepare peer researchers for research tasks. Our research protocol must first be described, since recommendations have indicated that the goals and content of training programs should be customized to concur with each context specificities [15].

## Methods

### Defining research protocol and recruiting peer researchers

#### Research objectives

Our study aimed to generate a questionnaire that could evaluate safety issues relevant to hospitalized patients experience, and to test it with them. “Patient experience” is defined as “the sum of all interactions, shaped by an organization’s culture, that influence patient perceptions across the continuum of care.” (Beryl Institute). Therefore, measuring patient experience should be done using subjective data, but also, and more importantly, using factual data [16]. Once hospitalized patients safety experience assessed, peer researchers were expected to identify possible improvement actions which were then proposed to the hospitals units hosting the study. These determined whether these propositions were acceptable, feasible and sustainable. Measures which were selected by a majority of involved hospitals units were then submitted to Public Hospitals of Paris, who subsequently encouraged their implementation. Given the fact that PUH are currently in the process of building up “offices of patient experience and partnerships”, our study’s goal was also to allow SURs to acquire new methodological skills in order to be able to become research assistants within future projects linked with this new structure.

#### Organization and follow-up

The two SURs members of PUH supervisory board were recruited as study’s co-organizers. They were regarded as leaders both among SURs and within the PUH network [17]. Interestingly, the main reason PUH leaders agreed to be involved in the project was because they trusted the co-organizers. The one most available for the project was recruited as a research assistant (¼ full-time equivalent). The other SUR representative was recruited on temporary missions.

A steering committee was constituted and included the two co-organizers, PUH representatives (doctors, nurse executives) and three members of the academic team, including the main investigator/trainer. This steering committee convened on a quarterly basis to ensure that research objectives were being met.

#### Peer researcher profiles, recruitment conditions and tasks

Since co-organizers knew every SUR in the PUH network, they acted as “contact tracers” [18] in order to recruit the ten SURs who could best contribute to the research. Recruiting peer researchers is a key challenge; selecting them with precision rather than based on convenience is therefore recommended [19]. In order to improve the likeliness of research results reaching local hospital committees, SUR co-organizers decided peer researchers should be president or vice-president of a local User Committee. Thirty-two SURs out of 139 were concerned. Among these 32 SURs, they looked for the following characteristics: having volunteered to participate (and therefore having an interest in research and a strong sense of commitment), having demonstrated team working abilities, being ready to put aside their position as SUR on hold to fully act as researchers, and being able to identify two healthcare units that would agree to participate in the study. Ten persons matched with this profile.

The average age of selected peer researchers was 60 years (43–84). Only one out of the ten had previously led research interviews as SUR. Two others had led surveys as part of their master degree studies.

Their main role was being peer interviewer but their work was not limited to collecting data: they were also asked to help create study tools, since their insights could improve survey relevance and intelligibility [19]. Finally, they were expected to contribute analyzing and interpreting data in order to identify which new professional practices might be worth promoting. Stipend for participating in the research project was approximately €3000 net of tax for each peer researcher based on an average workload of 10 h per month for 9 months.

#### Collected data

Our study focused on patients who were hospitalized for relatively short stays (between 3 days and 3 months). Patients were to be approached as close to their discharge date as possible to ensure that their health condition was compatible with their participation. For the qualitative phase of our research, 20 hospitalized patients were recruited. The saturation point was sought. After receiving participants consent, peer researchers were to lead peer-to-peer interviews which, by their very nature, are very comprehensive [20]. Peer researchers transcribed and analyzed the interview(s) they led. The main investigator then provided a cross-sectional analysis, which peer researchers discussed and could amend. Factors which impacted patient safety, and which hospitalized patients could report, were collected and analyzed. Based on these results, a final questionnaire was then created collectively and used by peer researchers within 160 hospitalized patients. Answers to this questionnaire meant to give a better idea of these factors frequency in order to identify priority measures to improve patients care safety experience. For example, time needed to respond to a patient’s buzz was often perceived as too long. Hence, peer researchers proposed PUH to establish two kinds of buzz: one for an emergency request, another for a non-emergency request.

#### Research ethics

Our research protocol obtained a favorable opinion from an ethics committee (IRB n° 2018–85); the European Ethical Appraisal Procedure was also respected (RPDG n°20,190,305,175,718). Study ethical character was heightened by the fact that it was “done with” research subjects’ representatives [21]. This meant that the ethics committee had to accept that data collecting tools would be communicated to them as they were being developed and not before the committee had released its opinion. These tools actually constituted our research first results since they were elaborated with peer researchers. Questions had to be carefully considered to avoid inducing inappropriate anxiety. To address this, we decided to provide psychological support contact information to participants.

All these considerations highlight the importance of peer researchers’ skills, as well as the need for high-quality training to prepare them to become peer interviewers.

## Results

### Training peer researchers: how our training program was designed and organized

Our training design falls within the field of “design-based research” (DbR), which is known for its pragmatic and collaborative scope [22]. DbR follows a systemic methodological paradigm which consists in including the perspectives of all collaborators. Expectations held by PUH representatives and peer researchers in relation to our training were reviewed. PUH representatives needed to be reassured about peer researchers’ ability to run the study, when peer researchers needed to become confident about their efficiency. Consequently, our training was designed to bring legitimacy and self-legitimacy.

PUH wished to help peer researchers broaden their knowledge of patient safety. Various training themes were suggested to peer researchers. SURs expectations revolved around the following topics (in decreasing order of interest): care safety, qualitative data collection, protocol understanding, research ethics, collaboration with a healthcare team, and respect of a scientific framework. The training program was also adjusted to take into account their requests regarding assessment format, duration and procedures.

The main investigator/trainer talked about some of the methodological challenges that could occur in participative studies [23] such as priorities based on unsound criteria [24], inclusion criteria not respected, uncompletion of expected procedures [25, 26]. Peer researchers were asked to differentiate between the experience of safety and the feeling of safety, to hone their understanding of inclusion and exclusion criteria, to understand the logics inherent to the different phases of research (from the academic journal to the focus group, to the collection of qualitative and quantitative data) and to prepare them for data analysis. Each of these aspects was tailored to our research project and all of our training steps occurred in the same sequence as our research project steps. This training strategy was chosen for 3 reasons: to ensure optimal motivation of peer researchers for information provided, to increase understanding and memorization through immediate application, and to make sure that practice might complete theoretical learning (learning by doing).

Several evaluations were conducted: a questionnaire was distributed before the training to observe how participants feeling of efficiency evolved, as well as to see how their practical knowledge of research progressed. For each participant, training program results were assessed through a coding system in which user anonymity was also respected.

Debriefings took place after each training session to check whether the content was relevant and if it had been understood. They were conducted at the end of each day to ensure that participants still recalled events clearly. Participants were given an anonymous assessment sheet in which they were asked to estimate each workshop usefulness (poor, average, good, excellent) and to rank their overall satisfaction (grades ranging from 0 to 5).

### Practical modalities and examples of training program educational content

Peer researchers were compensated for their participation in the training program as well as for all other phases of our research. Our training program lasted 45 h over the course of 10 days (Table 1). Forty-two of these hours were in-person training. PUH provided facilities and materials. Sessions took place at the same pace as the actual research project; training sessions therefore were held over a six-month period. Extra sessions were scheduled in the case of absences, as recommended [14]. Two people benefited from these catch-up sessions at the very beginning of the training.

**Table 1:** Training program organization and overall content (48 h)

Teaching Goals	Sources for Teaching Goals	Contents	Teaching Techniques	Trainers
DAY 1 : 7 in Person hours				
Getting familiar with the protocol, clarifying each person’s role	Elliott et al., 2002 [27]; Brett et al, 2012 [20].	Phases, inclusion criteria	Interactive presentation (questions and answers)	Main investigator
Knowing scientific norms	Westfall et al., 2017 [15]	Reliability, coherence	Interactive presentation (questions and answers)	Main investigator
Identifying adverse events	At the promoter’s request; Westfall et al.,2017 [15]	Risks in terms of hygiene, identity vigilance, medicine, etc.	Presentation, theme-based study of complaints	Public Hospitals of Paris, peer co-organizers
Identifying causes of adverse events	At the promoter’s request; Westfall et al., 2017 [15]	Human errors, organizational errors, differentiating between mistakes and fault	Videos on analysis of feedback	Public Hospitals of Paris
Getting familiar with ethical norms	Westfall et al., 2017 [15]	Consent, confidentiality	Case studies, questions and answers	Main investigator
DAY 2: 7 in-person hours				
Understanding the purpose of collaborative research projects		Results from studies, concept of empowerment	Interactive presentation (questions and answers)	Main investigator
Self-legitimizing, identifying the tensions inherent to the peer researcher position	Gross et al.2017 [23] ; INVOLVE; Devotta, 2014 [13]	Results from the research	Interactive presentation (questions and answers); reflexive exercises	Main investigator
Creating an interview guide		Based on the results from the focus group	Workshops in small groups	Peer co-organizers
DAY 3: 4 in person hours				
Collecting qualitative data	Elliott et al., 2002 [27]; Allam et al., 2004 [28]	Saturation, mistakes to avoid	Interactive presentation (questions and answers) and role-plays	Main investigator and peer co-organizers
DAYS 4,5,7,9: 6 hours at a distance (conference calls)				
Creating good group dynamics, sharing information, identifying answers	An instance that revealed poor group spirit; Elliott et al. 2002 [27],; Nicolaidis et al. 2012 [29], Leese et al., 2018 [30]	Access to health care services, to patients, information on publication projects	Interactive exchanges	Main investigator and peer co-organizers
DAY 6: 4 in-person hours				
Analyzing qualitative data	Faulkner, 2017 [31]	Theory, study of authentic complaints	Theoretical presentation; workshops in small groups	Main investigator
DAY 8: 7 in-person hours				
Making questions easier to understand, making questionnaire easier to handle		Going back on the creation process and examining the questionnaire	Interactive presentation (questions and answers)	Main investigator
Administering a questionnaire		Theoretical background and mistakes to avoid	Role-plays	Peer co-organizers
DAY 10: 8 in-person hours				
Analyzing and interpreting quantitative data	Jones et al. 2014 [17]	Results from the quantitative study	Workshops in small groups	Main investigator
DAY 11 : 3 in person hours				
Communicate research results			Role playing games	Main investigator

Once the research started, a support phase was implemented to answer questions and resolve any raised issues [27]. Discussions were held to determine whether this support phase should be offered only to those who were having difficulties. In an effort to avoid stigmatization, all peer researchers were invited to attend. For practical reasons, these sessions had to take place remotely; they took the form of regular updates during conference calls.

#### Content relating to patient safety

A certain number of issues regarding patient safety were presented to participants (differentiating mistakes from fault, identifying risk factors, reconstituting a chain of events, etc.). The point was to identify causal chains of undesirable events and to identify safety breaches. Differences in points of view between PUH trainers and peer researchers became apparent in the discussion, especially regarding how to categorize errors. For example, the case of a patient dying because of an allergic reaction to his roommate’s medicine which had been administered to him by mistake, was viewed as a medication error by PUH trainers and as a case of mistaken identity by peer researchers. Such gaps between the two perspectives are useful, as they highlight the fact that peer researchers interpret facts differently from professionals.

#### Content relating to self-legitimacy

A section of our training program was dedicated to helping participants self-legitimize as peer researchers. Participants were therefore asked to define the notion of “peer researcher” and to determine which factors made their contributions distinctive. Their input was supplemented with data from published literature to help them realize how their proximity with those who were being interviewed brought added value to research.

Trainers made sure that participants felt comfortable mobilizing their own experience. This meant that they could express themselves differently from academic researchers and that they could react more openly to respondents’ comments [13]. But, just like any other researcher, they should not infer someone else’s knowledge based on their own knowledge. They could, however, be confident using their own experience to better capture the experiences that they aimed to collect and assess. The following observations ensued: academic researchers have been known, at times – and more or less sincerely – to act as if they belonged to a particular social group in an attempt to better study it [32]. Thus, peer-researchers, who actually belong to the particular social group they were to study, were allowed to assume this position. This was emphasized to help them realize that they had particular strengths as peer researchers: they could improve data quality by increasing respondents’ levels of honesty [20], and by improving respondents’ comfort [27], all at the service of the relevance of results interpretation [20]. They were therefore advised to disclose that they were peers during research interviews without elaborating on their own experience of healthcare.

#### Content relating to scientific norms of research

Each step of our research was broken down into a precise set of actions wherein each action was discussed and justified. This was done to counter the risk of peer researchers establishing priorities based on unsound criteria [24]. Discussions revealed that peer researchers really needed to understand meaning behind each action before being ready to apply it. Answers to the evaluation questionnaire highlighted that peer-interviewers did not think that they should tape-record interviews if the respondents did not want them to do so. The research team had to justify this practice by explaining how these recordings were useful for the reproducibility of results.

#### Content relating to research ethics

Reports have stated that teaching ethical norms to peer researchers is more important than anything else [14]. Frequent ethical disagreements between research teams and peer researchers have been reported [21]. The notions of scientific integrity and responsibility towards those participating in the research was explained using the following statement: “That which is not scientific is not ethical and that which is not ethical is not scientific”. Certain elements had to be repeated several times before they were fully assimilated, namely notions related to confidentiality and, more specifically, the act of preventing people from being recognized regardless of the anonymization of data. Peer researchers had difficulties identifying which conduct to adopt if a problem were to emerge during the interview. It was hard for some of them – probably because they also happened to be user representatives – to give up solving problems directly by calling unit managers where patients were hospitalized. We also had to highlight the importance of not promising positive research impacts to those who were interviewed.

#### Content relating to the creation and appropriation of the interview guide

Our research protocol dictated that peer researchers create research tools under the main investigator guidance. Part of the training was therefore dedicated to this task. Co-organizers and the main investigator convened a focus group composed of peer researchers before the start of the training program. This focus group was meant to identify issues at stake in terms of care safety, especially those that interviewed patients were likely to mention, thus giving directions to build our interview guidelines. In addition to this analysis, the main investigator conducted a review of the literature to heighten data plausibility. The care safety issues that emerged from this review were added to the focus group results if their relevance made sense to peer researchers. One of the exercises was designed to make peer researchers become familiar with these issues, and for them to identify questions that would be open-ended enough to let the respondents generate new issues. The exercise also aimed to find ways of phrasing questions in positive (rather than negative) ways in order to avoid generating anxiety among those being interviewed.

Allowing peer researchers to understand the purpose of the preliminary work done in focus group was also important since these results were not used as directly as initially thought. The various points of view that emerged on this occasion were also an opportunity to remind peer researchers to keep an open mind and to welcome – even to seek – opinions that differed from theirs.

### Assessing our training program

Evaluations conducted throughout our training showed that participants found the course content to be very useful and that it helped them improve their research capacities. The knowledge tests they took are proof of this: participants came to understand that social science research could generate risks for those who were interviewed. Furthermore, before the training began, eight out of ten peer researchers felt like they knew precisely what could affect a patient’s experience of hospital safety; after the training (and before collecting the data), only two people out of ten still had such beliefs. This result shows that their attitudes evolved and that they learned to adopt a research-based approach. Consequently, the objectives of the training program were reached.

Furthermore, from one session to the next, it was clear that participants felt an increasing sense of responsibility towards the project. Perhaps more significantly, their adherence to the research project was excellent, as can be seen from their ongoing attendance during training sessions and from the ways in which they effectively contributed to research.

Although we have presumptive assumptions about this, it is impossible to know whether peer researchers succeeded in gaining more respondents or better data than academic researchers might have collected. But their data collection proved to be very useful: it improved the quality of their interpretations, which are clearly original when compared with scientific literature on care safety.

### Reviewing the encountered challenges and their possible resolutions

Before gaining the excellent results reported in III-3, a certain number of challenges had to be overcome. Rules for action (Table 2) are suggested based on the solutions developed in our research.

**Table 2:** Main observations

1. **Plan peer researchers recruitment.** Due to the fact that the project is not necessarily a priority for all involved or because of unforeseen events, it is necessary to have back-ups for key posts and to identify potential participant substitutes. 2. **Recruit peer researchers** based on their ability to listen (to the people being interviewed, to trainers and guidelines), as well as on their ability to question themselves, to pro-actively suggest solutions and to be able to handle moments of uncertainty. 3. **Design the training program** by combining the expectations of peer researchers with those of other persons involved in the project. 4. **Plan training** so that it happens synchronously with research steps. In this way, knowledge acquired in training sessions can be implemented directly. Needs can also be answered as they emerge. 5. **Improve one’s sense of ownership towards the project** through weekly meetings, whether in-person or long distance (as phone or video conference calls). 6. **Think about what motivates peer researchers.** Take their contributions into account, provide feedback as soon as they report information or make a comment, include them in the process of broadcasting the results, and encourage them to spread the results on their end. 7. **Develop a relationship between academic researchers and peer researchers based on mutual trust and respect,** with the understanding that peer co-organizers help facilitate this effort. 8. **Share control over research.** Academic researchers must demonstrate an ongoing ability of being reflexive in order to include peer researchers’ contributions, and to reconsider their own research practices. 9. **Plan ways to value peer researchers:** through financial compensation, but also by recognizing their skills (especially by giving them university ECTS credits recognizing their training).	

#### Challenges relating to project organization

Very early in our training, one of the selected peer researchers had to withdraw from the project due to serious family matters. In addition, during the study, one peer researcher resigned. Offsetting peer researchers’ turnover [33] had been anticipated beforehand; a waiting list had therefore been arranged, which allowed these peer researchers to be replaced immediately. As soon as the substitutes were recruited, they attended one-on-one training sessions to make up for the group trainings they had missed.

To counter the fact that some peer researchers did not have access to the Internet, certain logistical resources and a sense of adaptability had to be mustered. Our training program lasted longer than initially planned because participants asked for more time. The importance of a training program’s duration and quality was highlighted throughout this experiment when preparing SURs for their roles as peer investigators and helped to create a positive group dynamic around the research project.

#### Challenges relating to peer researcher’s involvement

The involvement of peer researchers revealed itself to be both major and insufficient, as had been the case in other studies [34]. Peer researchers’ participation in the training was diligent and excellent. When the project started, they declared that their involvement was not driven by financial motives. But the project was nearly halted when they realized that they would have to submit to university’s administrative demands in order to receive their stipend; they contested having to go through this process in light of their particular situation. This incident highlighted once again their need to attribute meaning to all of their actions. Furthermore, the university announced that it was unable to employ people who were older than the legal working age limit, leading some peer researchers to cast doubts on their participation in the project. While alternative solutions were being sought, some peer researchers trust in the research team decreased, with emphasis being put on the team’s lack of foreseeing on this issue. While the innovative nature of this research and the approach that consisted in advancing by trial and error had been openly discussed, the uncertainty inherent to the process was, for some peer researchers, difficult to understand at the very beginning of the process.

Training program have been described as a good time to foster a sense of inclusion among research group members and a way to keep this connection alive throughout the research process [34], but it was not that simple. The main investigator/trainer tried to step back to let peer researchers take ownership of the research. But, implicitly, they sought validity in their actions and thought that the main investigator/trainer would guide them step by step. Co-organizers played a major role as they were given the task of managing the research on a daily basis while the investigator focused on managing purely methodological issues. The idea of a regular collective phone call was suggested to generate better group cohesion. Once implemented, this solution worked very well. These exchanges served as a framework for the section initially planned as “practical support”.

#### Challenges relating to recruitment aspects

Co-organizers started doubting their own recruitment methods when one of the peer researchers resigned and when faced with a rather heterogeneous commitment level of peer researchers. The research team, including the co-organizers themselves, questioned themselves on their decision to delegate the task of recruiting peer researchers to the two co-organizers. They wondered whether they should have conducted several focus group sessions with a larger panel of user representatives; this would have allowed them to identify adequate profiles, rather than solely rely on intuition and affinity.

It had been decided not to rely on recruitment interviews to avoid hurting unselected SURs and generate lasting resentment against the two co-organizers with whom they are colleague in different instances. If recruitment had been made by the main investigator, this would not have been of consequence, but peer researchers’ commitment to the project would have undoubtedly been weaker.

In retrospect, at the recruitment phase, nothing distinguished peer researchers who ended up being the most involved in research. All of them seemed equally eager and interested. Some were expressing a higher feeling of self-efficiency than others, but this did not translate into better performance. Nevertheless, two main peer researcher qualities emerged as keys to success: a critical mind and an ability to listen and from then, learn new skills. But, in the end, our experience calls for recruiting more peer researchers than initially needed, to compensate for casting errors and turn-over, whatever their reasons.

#### Emotional challenges faced by academic researchers

In collaborative research, academic researchers are in charge of the investigative process. In our project, such a responsibility proved to be burdensome since the academic researchers sometimes doubted the project’s feasibility. This occurred mostly when peer researchers’ involvement was still subject to fluctuations, but it was also related to peer researchers’ interviewing skills. For example, the main investigator/trainer indicated, as she usually does in master classes, that research interviews should be conducted as conversations rather than interrogations. However, role-playing games revealed that peer researchers tended to veer away from interview guidelines and elicited conversations that were only loosely connected to the research. This highlighted how important it was to provide training material that differed from the one targeted at academic researchers, as these tend to struggle more with leaving frameworks rather than entering them.

Finally, it was difficult to delegate tasks for which we were perceived as responsible. While this may not be specific to participative studies with SURs, it may have heightened the issue.

In retrospect, the biggest difficulty may have been to accept that our assumptions – including scientific positions – would be challenged. For participative research to be meaningful, it has to include co-researchers’ input. In the context of this study, this stance led us to reconsider the consent and information forms. Peer researchers did not relate to the initial material, which they viewed as “boring” and “filled with jargon”. They suggested other options which were more “catchy” than what usual academic standards allow. Academic researchers had to accept these types of proposals and welcomed the idea that the second text was more engaging, even if, as researchers, they tended to avoid using a motivational tone for such documents. However, other requirements were non-negotiable, such as the normative requirements for procedural ethics, which had to be included in the text, even if peer researchers found that some sentences were too heavy-handed.

## Discussion and limits

This paper deliberately focuses on the research process rather than on the results of our research. This was done to respond to the observation stating that implementing user engagement is still a complex task [35]. Three logics guided the research project and therefore the training’s elaboration: a power focus one which aims to rebalance power relations [36]; a priority setting one which aim to involve patients in setting research priorities [36] and an epistemic one which aims to ensure that the research benefits from the inputs of the co-researchers. This guided the training which have been conducted in sync with the research process. It has covered all possible stages in participatory research [37]. Peer researchers learned to conduct research largely *by doing it* which is not unlike what happens with a more academic audience. It alternated between theory-based sessions, practical application phases and reflexive analysis. Finally, the co-researchers really took ownership of the project thanks to the frequency of the meetings and the development of their sense of competence. The research process has empowered them as they lead it. It has optimized their ability to become agents of change: in particular they learned to present their research results in an academic’ manner and this has maximized their voice. But what happens after the research is over remains a gap that it would be worth being investigated. It would be interesting to follow the co-researchers’ evolution after this research: will they be able to generate new research opportunities to get involved in? Indeed, ethically, it is considered as essential to initiate empowerment actions if, and only if, there is a chance they may continue beyond the study [38].

The training model presented here was designed as a global process; we therefore did not attempt to find out whether some phases of our training program had been more successful than others. Finally, the research team made nine observations which were formulated as recommendations emanating from the research. Since these elements were identified empirically, future studies have yet to confirm our results.

## Conclusion

Our research confirms the many studies that have concluded that participative research in the context of hospital care is feasible. Furthermore, we have established that service user representatives can become peer researchers if they undergo specific training. They must learn, among other things, to adhere to notions that are essential to research work, such as confidentiality and the non-disclosure of results during an ongoing study. They must also acquire a specific set of skills, preferably in group settings, since groups contribute to cope with diverse opinions. This, in turn, contributes to a positive attitude towards learning and completing research. A relatively intense training tends to reassure each party, including peer researchers themselves, on the ability of everyone involved to implement the research project. Furthermore, highly experienced trainers are needed to conduct training sessions, since the learners were particularly eager to attribute meaning to their learning. The success of such a project also depends on mutual trust among peer researchers. Academic researchers are required to have emotional, adaptive, and, in certain cases, negotiation skills. Finally, our training program was a two-way process as both trainers and trainees were ready to learn from each other and to reconsider their own assumptions. Academic researchers, in particular, had to be ready to learn from peer researchers, to let themselves be surprised and to allow their own research practices to evolve.

## Data Availability

Data sharing is not applicable to this article as no datasets were generated or analyzed over the course of the study.

## Abbreviations

SURs: Services User Representatives
PUH: Paris University Hospitals
DbR: Design-based Research

## Footnotes

1 Law No. 2002–303 du 4 mars 2002 relative aux droits des malades et à la qualité du système de santé
